# Improving the capacity of teachers to teach Family Life and HIV Education (FLHE) in Nigeria through mobile technology: a randomized controlled trial

**DOI:** 10.21203/rs.3.rs-7012994/v1

**Published:** 2025-08-13

**Authors:** Emmanuel Adebayo, Adedamola Adebayo, Adesola Olumide, Adesola Ogunniyi, Wafaie Fawzi

**Affiliations:** University of Ibadan; Independent Consultant; University of Ibadan; University of Ibadan; Harvard T.H. Chan School of Public Health

**Keywords:** Adolescent Health, mHealth, Comprehensive Sexuality Education, Teacher training, Mobile application

## Abstract

Adolescents in Nigeria face several sexual and reproductive health issues, such as HIV/AIDS and other sexually transmitted infections. To help address these problems, the government introduced a school program called Family Life and HIV Education (FLHE). This program is meant to provide adolescents with important information and skills to protect their health. However, many teachers do not receive enough training to deliver the curriculum effectively, mostly due to limited resources and the high cost of in-person training. This study tested whether using mobile phone apps could help train teachers more efficiently. We worked with secondary schools in Ibadan-North, Oyo State, and divided them into three groups. One group received in-person training, another used a mobile app for training, and the third group received no training. The study looked at how much teachers learned and how confident they felt about teaching the curriculum. The results showed that both the in-person and mobile app trainings helped teachers improve their knowledge and teaching confidence. However, teachers who used the mobile app were better at retaining what they learned over time compared to the other groups. This suggests that mobile technology can be just as effective as traditional training and possibly even better for long-term learning. It also has the advantage of being easier to scale up, especially in areas with fewer resources. This study supports the use of mobile learning tools to improve how teachers are trained to deliver sexuality education in Nigeria and similar contexts.

## Background

Adolescents represent a significant proportion of the global population, particularly in low- and middle- income countries, where they often face substantial health and developmental challenges (1,2). Sexual and reproductive health issues, including HIV/AIDS and other sexually transmitted infections (STIs), remain prominent concerns for this age group(3,4). Educational interventions, particularly through schools, are a critical avenue for addressing these challenges. Comprehensive Sexuality Education (CSE), which integrates information on human development, relationships, life skills, and health, has been identified as a key strategy for equipping adolescents with the knowledge and skills they need to make informed decisions about their sexual and reproductive health(5,6).

In Nigeria, the Family Life and HIV Education (FLHE) curriculum was introduced in 2003 by the Federal Ministry of Education as a school-based HIV prevention intervention. It was designed to provide adolescents with critical health education through trained teachers(7). Despite the proven effectiveness of the FLHE curriculum (8,9), significant challenges have hindered its full implementation (10). A major barrier is the inadequate training and capacity of teachers to deliver the curriculum effectively (11). Traditional face-to-face training approaches, though widely used and effective, are resource-intensive, time-consuming, and difficult to scale, particularly in resource-constrained settings(12–14). Furthermore, the sustainability of knowledge and skills gained through these methods is often limited. Recent advancement in digital technology offers promising alternatives for addressing these challenges. Digital tools, including mobile applications and online platforms, provide opportunities for scalable, flexible, and potentially more sustainable teacher training solutions (15–18).

Mobile-based interventions have gained traction as an innovative approach to addressing systemic barriers in education and healthcare delivery(19–21). Studies have shown that technology, such as virtual reality, online platforms, and AI, can significantly enhance teacher training by providing innovative and interactive learning experiences (17). Digital tools offer several advantages, including scalability, flexibility, and interactivity, which can bridge the gaps in teacher training and resource accessibility. Studies have also shown that use of digital tools in teacher training can improve teaching effectiveness of teachers (ref). A systematic review concluded that interactive technologies hold substantial potential in transforming teacher education in Africa (22). These findings collectively underscore the potential of technology, including mobile-based technology, to transform teacher education generally, including for CSE.

Building on this growing body of evidence and considering the challenges in teacher capacity in the delivery of CSE, this study sought to assess the efficacy of digital tools in improving teachers’ knowledge and capacity to deliver the FLHE curriculum in school. Using a randomized controlled trial (RCT) design, teachers were randomized into one of three groups: a traditional face-to-face group (standard group), a digital tools-based training group (experimental group), or a control group. By comparing the outcomes across the three arms, the research aimed to provide robust evidence on the comparative effectiveness of mobile-based and traditional face-to-face training approaches.

This study is particularly timely given the growing reliance on digital solutions in education and public health. By addressing critical gaps in teacher training, the findings are expected to inform the design of future intervention that harness mobile technology to strengthen FLHE delivery in Nigeria and beyond.

## Methodology

This study was designed to compare the effectiveness of the traditional didactic face-to-face approach and the use of a mobile application on the knowledge, and self-reported capacity to teach Family Life and HIV Education among teachers.

### Study design

This study employed a multi-phase, cluster-randomised controlled trial (RCT) design to evaluate the efficacy of a mobile application in enhancing the capacity of teachers to deliver Family Life and HIV Education (FLHE) in secondary schools in Ibadan-North Local Government Area (LGA), Oyo state, Nigeria. The trial was registered at the Pan-Africa Clinical Trial registry (PACTR202206840350306). Details of the methodology of this study have been published in another paper(23).

This study was conducted in three phases: phase one surveyed eligible schools and collected information from stakeholders on the feasibility of a mobile app in building the knowledge and capacity of teachers. Phase two involved the development of the mobile application and the adaptation of the FLHE teachers’ training curriculum for the mobile application. The evaluation questions for this study were also pretested during phase two. Phase three involved the implementation of the intervention. Evaluation of the study was designed to be conducted pre-intervention, immediately after the last training session (assessment 1), one-month after (assessment 2), and three-months after intervention (assessment 3).

### Participants

Participants for this study were teachers in public/government funded junior secondary schools in Ibadan North Local Government Area. A total of 46 teachers per arm was calculated as minimum sample size for this study. Using a proportion of teachers who felt students were too young to be taught about sexuality and HIV in a previous study(24), assuming a minimum difference of 30% between pre and post intervention proportions, and adding 10% for loss to follow up, an approximate number of participants per group was derived.

Eligibility criteria for schools:

1. The school must be a public/government funded schools
2. Have knowledge of the FLHE program, with the head of school able to confirm that the teachers are aware of FLHE being part of the curriculum.
3. The schools must have at least 10 eligible teachers that can participate in the study
4. The head of school must consent to the study and willing to allow teachers participate in the study

Eligibility criteria for teachers:

1. Teachers currently employed in government – owned (public) secondary schools within the selected LGA.
2. Teachers teaching at least one subject designated as part of the FLHE curriculum.
3. Certified, experienced (at least one year teaching the course/subject), and full -time teachers.

### Intervention procedures

The details of the intervention procedures are published in a previous methodology paper. This trial had three arms: control, standard, and mobile app groups. The control group received a one-time in-person training on how to communicate with adolescents. The standard group received a four weeks (four hours a week) in-person training sessions on FLHE. The mobile app group received an initial one-time in-person training on how to use the mobile app, followed by a four-week online training on FLHE through the mFLHE app designed.

### The Mobile application

The mFLHE mobile app was developed to facilitate structured educational engagement among the teachers (participants) and between teachers and moderators by providing a platform for accessing curriculums, taking assessments, and enabling communication. The app was specifically designed for Android devices and requires limited internet connectivity to function effectively. It allows participants to access weekly curriculum content, engage in discussions, download lesson documents (for offline access), and communicate in real-time with both peers and moderators. Moderators, on the other hand, could create and manage weekly topics, upload relevant curriculum documents, broadcast information, and monitor participant engagement. Moderators have additional administrative capabilities such as creating and managing participant accounts.

Functional features included user account creation, authentication, week and subject configuration, document upload and viewing, real-time chat, and push notifications. The development stack consisted of Flutter and Dart, with Android Studio serving as the primary IDE. An Agile development approach was used, focusing on iterative sprints and feature-driven implementation. For state management, the Bloc pattern was employed to maintain predictable and scalable handling of user interactions and application state. The development workflow involved converting UI/UX prototypes into functional Flutter interfaces, implementing navigation logic, creating API service layers, and integrating these components to ensure seamless user experience.

As part of the quality assurance process, standard testing procedures were carried out to ensure the app met both functional expectations and industry best practices. Each feature underwent unit and integration testing to validate performance and stability. Firebase Crashlytics was used to detect and log crashes, enabling thorough debugging and verification before features were marked as complete. During this process, an early challenge was encountered when the 1GB daily download limit on Firebase’s free-tier cloud storage was exceeded, resulting in failed document downloads. This was resolved by enabling Firebase billing and introducing a bandwidth control strategy, which involved disabling access to future weekly content until it became relevant.

To maintain compliance with standard mobile development practices, efforts were made to secure user data, handle errors gracefully, and ensure responsive UI performance across supported devices. Additionally, since the app was not published on the Google Play Store (to restrict access during the research period), updates were not distributed automatically. Instead, updated APKs were stored on a secure file server, and download links were shared with users to manually install new versions. This manual update process ensured that post-deployment bugs could be addressed promptly, while also allowing for controlled rollout and continuous improvement of the app.

### Data collection

#### Measurement of variables

1. Knowledge of FLHE: This variable was assessed using a 20-item true/false scale. Each correct answer was awarded one point, while incorrect answers received zero points. The highest possible score was 20 points. A cut-off score of 50% (10points) was used to categorize knowledge levels. Scores of 10 and above indicated good knowledge, whereas scores below 10 were designated as poor knowledge.
2. Self-reported capacity: This variable was measured using a 9-item scale, with responses graded from “Always” to “Not Sure” (Always, Sometimes, Never, Not Sure). The highest obtainable score was 18 points. A cut-off score of 9 points was used to differentiate between good capacity and poor capacity.

## Data analysis

In this study, a comprehensive statistical analysis was conducted to examine the data. Descriptive statistics, including frequencies, percentages, and means, were calculated to summarize the characteristics of the participants. For inferential analysis, both bivariate and multivariate techniques were employed. Bivariate analysis was performed using chi-square tests, T-tests, and ANOVA to explore associations and differences between variables. Additionally, logistic regression analysis was conducted to identify predictors and assess for significant differences while controlling for confounders. These methods provided a robust framework for understanding the relationships within the data and drawing meaningful conclusions from the study findings.

### Ethical considerations

The study received approval from the Oyo State Ministry of Education. Additionally, permission was obtained from the Association of School Principals in the state. Ethical approval was granted by the Oyo State Research Ethics Review Committee (approval number: AD/13/479/4276^B^) and the University of Ibadan/University College Hospital Ethics Review Committee (approval number: UI/EC/21/0521). Following these approvals, eligible schools were approached. The head teacher (principal) of each school was briefed on the study’s purpose and given the opportunity to consent to their school’s participation. The principals for each of the selected/consenting schools were blinded to the group assignments. Participants provided informed consent after the trial details were explained to them. Teachers were also blinded to the involvement of other groups.

## Results

### Participant characteristics

Table one presents the socio-demographic characteristics of participants across the groups across the data collection period. Participants across the groups were mostly similar throughout the course of the study. At inception, there were 47, 48, and 50 participants recruited to participate as control, standard and mobile groups participants. This remained largely the same across the period of the study, except for one participant that was lost to follow up after the intervention period. Further, 3 participants in control group also missed data collection at different points after the initial data collection. The data from these three participants were not included in the data analysis.

### Knowledge of FLHE

Table two shows the proportion of participants with good knowledge of FLHE. Knowledge was categorized as good or poor using the mean score within the group as the cut-off point. Highest obtainable score was 20. Within each group there was significant increase in knowledge among standard and mobile group participants in the immediate post intervention period. However, mobile group participants had significant increase in knowledge one month and three months after intervention. Across the groups, in comparison to control groups, mobile participants reported significantly higher knowledge scores one month and three months after intervention (Table 3).

As compared to baseline scores, the capacity of all participants increased significantly at one-month post intervention. However, while this capacity of participants in control group decreased at three-months post intervention, the capacity of standard and mobile group participants increased was still significantly higher at three-months as compared to the post intervention (Table 4). Across the groups, as compared to the control group, the self-reported capacity of participants in the standard and mobile app group were significantly higher at three-months post intervention (Table 5). There was no significant difference in the self-reported capacity of standard and mobile group participants across the period of the study.

## Discussion

This study assessed the effectiveness of using mobile technology to improve teachers’ knowledge and capacity to deliver FLHE compared to the traditional face-to-face training method. The findings provide valuable insights into the potential of digital tools as an alternative to traditional teaching methods, especially in resource-low settings.

### Knowledge of FLHE across groups

There was significant increase in knowledge among participants in both the standard and mobile app groups as compared to the control immediately after the intervention. However, knowledge was significantly higher among standard group as compared to mobile app group. Notably, while knowledge declined slightly over time in both intervention groups, the mobile app group maintained significantly better knowledge retention at one-and-three-month post intervention compared to the control. This suggests that mobile technology has the potential to sustain knowledge over a longer period, due to its flexibility and the fact that participants can revisit materials at their own time and pace.

The sustained knowledge in the mobile app group can be attributed to the accessibility of the app, which allowed participants to review content repeatedly at their convenience. According to the constructivist learning theory (CLT), learners can build their understanding through active engagement with content(25,26). The continuous engagement with content may have helped participants build their knowledge. The mobile app encouraged teachers to actively engage with the FLHE curriculum at their own pace. The interactive elements of the app (discussion forums, quizzes) supported experiential learning, aligning with the CLT. This study provides evidence that digital tools can enhance learning by fostering interaction and active participation. In contrast, the decline in knowledge among the standard group participants over time may highlight the limitations of the traditional didactic approach, where once the training ends, opportunities for reinforcement are minimal. Published reviews have highlighted the fact that one of training does not work in improving the capacity of teachers to teach FLHE (27). These findings underscore the need for ongoing engagement and the potential value of digital tools in addressing this gap.

The significant difference between the intervention groups at immediately after intervention and the sustained knowledge among participants in mobile app group, show a complimentary opportunity. While the face-to-face approach may be better at providing participants with significantly better knowledge, the mobile apps have the unique ability to sustain knowledge among participants. Studies have shown that a blended approach could yield a more significant results than either online or face-to-face approach alone (18,28).

### Self-reported teaching capacity

Self-reported capacity to deliver FLHE improved significantly in both the standard and mobile app intervention groups, with no significant differences between the two. This suggests that mobile applications are equally as effective as traditional face-to-face training in building teachers’ confidence and perceived capability to deliver FLHE. A similar finding has been reported in studies that utilised digital platforms or online means to build capacity of teachers (15,29–32). A 2023 systematic review comparing the effectiveness of face-to-face versus online delivery of continuing professional development for science teachers reported that there is a comparable effectiveness between use of online media and face-to- face. The review reported that the effectiveness of both modes was largely influenced by similar external factors(31).

Despite the similarities in capacity outcomes, the mobile app group’s consistent performance overtime suggests that mobile tools may provide more consistent reinforcement of training concepts. The sustained increase in capacity, despite slight knowledge declines, suggests that hands-on practice and interaction with the training materials could have played a crucial role in reinforcing participants’ confidence. Teachers in both intervention groups may have had opportunities to apply what they learned in their teaching environments, leading to the consolidation of skills and capacity over time. This finding highlights the importance of practical application in translating knowledge into confidence and capability. Studies have shown that while face-to-face training events may be more valued and perceived as having better quality and engagement(16,33), online communities provide valuable resources and support for curriculum issues and can be more effective in conceptual understanding and overall teacher satisfaction(29,33).

### Implications of the findings

The result of this study adds to the body of literature that confirms that mobile app-based training method is not only effective but also offers unique advantages over the traditional training approach. The accessibility and self-paced nature of the mobile app allowed participants to learn at their own convenience, which may have contributed to the better knowledge retention observed in the mobile group. Further, the digital platform’s multimedia capabilities (the audio-visual content) was a unique feature in the app tested and may have likely enhanced engagement and comprehension. The inclusion of a discussion forum also allowed for peer-to-peer learning, allowing for further discussions and engagement with the materials.

The mobile app’s comparable effectiveness to face-to-face training has significant implications for scalability and sustainability. Mobile technology can overcome logistical challenges such as limited infrastructure, travel costs, and scheduling conflicts, which often hinder traditional training methods(13,14). This is particularly relevant in resource-constrained settings like Nigeria, where access to consistent training opportunities is limited.

The findings have several broader implications. Mobile applications offer a scalable and cost-effective solution to teacher training, particularly in regions where traditional workshops are logistically challenging. The adaptability of mobile platforms allows for the inclusion of culturally relevant materials and supports learning at the teachers’ convenience. These tools also enable ongoing professional development by providing refresher modules and updates, addressing the issue of knowledge decline observed in this and other studies.

### Limitations and Future Directions

While the study highlights the effectiveness of mobile technology, it is important to consider potential challenges. Participants in the mobile app group may have benefited for the novelty of the digital platform, which could have influenced their engagement and motivation. Long-term studies are needed to assess whether the observed benefits are sustained over extended periods.

Additionally, while this study focused on knowledge and self-reported capacity, future research could explore the impact of these training methods on actual teaching practices and student outcomes. Understanding how these approaches influence classroom delivery and adolescent learning will provide a more comprehensive evaluation of their effectiveness.

In this study, participants were provided with mobile phones and internet services, highlighting the need for future research to assess the real-world application of these methods for scaling purposes. Conducting implementation research to evaluate the scalability of these approaches is essential.

## Conclusion

Conclusively, this study provides strong evidence that mobile technology is a viable and effective alternative to traditional face-to-face training for improving teachers’ knowledge and capacity to deliver FLHE. The sustained knowledge retention observed in the mobile group highlights the unique advantages of digital tools in promoting long-term learning. As education systems increasingly seek scalable and innovative solutions, mobile technology offers a promising pathway for enhancing teacher training, particularly in resource-constrained settings. Future interventions should focus on integrating periodic refresher training and exploring the broader impacts of these methods on educational outcomes.

## Figures and Tables

**Table 1:** Participants’ socio-demographic Characteristics

Groups	Subgroup	Pre-intervention	Immediate post-intervention	One-month post-intervention	Three-months post intervention
Control*		47	47	46	46
Standard*		48	48	48	48
Mobile		50	50	50	50
**Gender**					
Control	Male	19	19	19	19
	Female	25	25	25	25
Standard	Male	15	15	15	15
	Female	33	33	33	33
Mobile app	Male	17	17	17	17
	Female	33	33	33	33
**Have Teenage child**					
Control	Yes	31	31	31	31
Standard	Yes	28	30	30	31
Mobile app	Yes	32	32	32	32
		Mean (SD)	Mean (SD)	Mean (SD)	Mean (SD)
**Age**	Control	44.8 (8.3)	44.8 (8.5)	44.8 (8.5)	44.8 (8.5)
	Standard	42.0 (7.9)	42.2 (7.8)	42.2 (9.6)	42.7 (7.8)
	Mobile app	43.2 (9.9)	43.2 (10.1)	43.2 (10.2)	43.5 (10.0)
**Average number of classes per week**	Control	17.38 (7.9)	18.25 (8.6)	18.25 (7.2)	17.7 (7.3)
	Standard	16.7 (7.1)	17.0 (7.1)	16.9 (7.4)	16.8 (7.4)
	Mobile app	17.6 (6.3)	18.0 (6.1)	18.2 (6.2)	18.0 (6.9)
**Number of years in service**	Control	14.2 (9.9)	14.2 (9.9)	14.3 (10.2)	14.3 (10.2)
	Standard	10.9 (9.2)	10.9 (9.2)	11.8 (9.6)	11.8 (9.6)
	Mobile app	13.4 (10.9)	13.4 (10.9)	13.8 (10.7)	13.8 (10.9)

* Three teachers in control and two teachers selected to participate in standard groups did not show up on the day of the intervention.

**Table 2:** Knowledge of FLHE within groups across time

Group/ Intervention period		Pre-Intervention N(%)	Immediate post intervention N(%)	One month after N(%)	Three months after N(%)
Control	Good knowledge	17 (38.6)	26 (59.1)	22 (50.0)	21 (47.7)
Standard	Good knowledge	27 (56.3)	37 (77.1) *	31 (64.6)	30 (62.5)
Mobile App	Good knowledge	25 (50.0)	36 (72.0) *	35 (70.0) *	35 (70.0) *

* Significant at p < 0.05

**Table 3:** Knowledge Comparison between groups across time

Group	Pre-interventions	Immediate post intervention	One month post intervention	Three months post intervention
Control	1	1	1	1
Standard	2.04 (0.89–4.70)	2.3 (0.95–5.74)	1.8 (0.79–4.21)	1.8 (0.80–4.19)
Mobile group	1.6 (0.70–3.61)	1.8 (0.75–4.21)	2.3 (1.00–5.44) *	2.6 (1.10–5.96) *

* Significant at p < 0.05

Self-reported capacity to teach FLHE

**Table 4:** Self-reported Capacity

Group		Pre-Intervention	Immediate post intervention	One month after	Three months after
Control	Good capacity	11 (25.0)	19 (43.2)	20 (45.5) *	18 (40.9)
Standard	Good capacity	20 (41.7)	27 (56.3)	30 (62.5) *	32 (66.7) *
Mobile app	Good capacity	23 (46.0)	30 (60.0)	33 (66.0) *	33 (66.0) *

**Table 5:** Self-reported capacity Comparison between groups across time

Group	Pre-interventions	Immediate post intervention	One month post intervention	Three months post intervention
Control	1	1	1	1
Standard	2.1 (0.88–5.23)	1.69 (0.74–3.86)	2.0 (0.87–4.60)	2.9 (1.24–6.75) *
Mobile group	2.6 (1.06–6.16) *	1.97 (0.87–4.49)	2.33 (1.01–5.36) *	2.8 (1.21–6.49) *

## Data Availability

The datasets used and analysed during the current study are available from the corresponding author on reasonable request.

## Abbreviations

FLHE: Family Life and HIV Education
mFLHE: Mobile Family Life and HIV Education
RCT: Randomized Controlled Trial
LGA: Local Government Authority
ANOVA: Analysis of Variance
